# Spectral CT imaging of human osteoarthritic cartilage via quantitative assessment of glycosaminoglycan content using multiple contrast agents

**DOI:** 10.1063/5.0035312

**Published:** 2021-04-01

**Authors:** Kenzie Baer, Sandra Kieser, Ben Schon, Kishore Rajendran, Timen ten Harkel, Mohsen Ramyar, Caroline Löbker, Christopher Bateman, Anthony Butler, Aamir Raja, Gary Hooper, Nigel Anderson, Tim Woodfield

**Affiliations:** 1 Christchurch Regenerative Medicine and Tissue Engineering (CReaTE), Department of Orthopaedic Surgery and Musculoskeletal Medicine, University of Otago Christchurch, Christchurch 8011, New Zealand; 2 Medical Technologies Centre of Research Excellence (MedTech CoRE), Auckland 1010, New Zealand; 3 Department of Radiology, University of Otago Christchurch, Christchurch 8011, New Zealand; 4 MIRA Institute for Biomedical Technology and Technical Medicine, Department of Developmental Bioengineering, University of Twente, 7500 AE ENSCHEDE, The Netherlands; 5 School of Physical and Chemical Sciences, University of Canterbury, Christchurch 8041, New Zealand; 6 MARS Bioimaging Ltd, Christchurch 8041, New Zealand; 7 European Organization for Nuclear Research (CERN), Geneva 1211, Switzerland; 8 Human Interface Technology Laboratory New Zealand, University of Canterbury, Christchurch 8041, New Zealand; 9 Physics Department, Khalifa University, Abu Dhabi, Zone 1, United Arab Emirates

## Abstract

Detection of early osteoarthritis to stabilize or reverse the damage to articular cartilage would improve patient function, reduce disability, and limit the need for joint replacement. In this study, we investigated nondestructive photon-processing spectral computed tomography (CT) for the quantitative measurement of the glycosaminoglycan (GAG) content compared to destructive histological and biochemical assay techniques in normal and osteoarthritic tissues. Cartilage-bone cores from healthy bovine stifles were incubated in 50% ioxaglate (Hexabrix^®^) or 100% gadobenate dimeglumine (MultiHance^®^). A photon-processing spectral CT (MARS) scanner with a CdTe-Medipix3RX detector imaged samples. Calibration phantoms of ioxaglate and gadobenate dimeglumine were used to determine iodine and gadolinium concentrations from photon-processing spectral CT images to correlate with the GAG content measured using a dimethylmethylene blue assay. The zonal distribution of GAG was compared between photon-processing spectral CT images and histological sections. Furthermore, discrimination and quantification of GAG in osteoarthritic human tibial plateau tissue using the same contrast agents were demonstrated. Contrast agent concentrations were inversely related to the GAG content. The GAG concentration increased from 25 *μ*g/ml (85 mg/ml iodine or 43 mg/ml gadolinium) in the superficial layer to 75 *μ*g/ml (65 mg/ml iodine or 37 mg/ml gadolinium) in the deep layer of healthy bovine cartilage. Deep zone articular cartilage could be distinguished from subchondral bone by utilizing the material decomposition technique. Photon-processing spectral CT images correlated with histological sections in healthy and osteoarthritic tissues. Post-imaging material decomposition was able to quantify the GAG content and distribution throughout healthy and osteoarthritic cartilage using Hexabrix^®^ and MultiHance^®^ while differentiating the underlying subchondral bone.

## INTRODUCTION

Osteoarthritis (OA) is a common cause of disability and the most common indication for both knee and hip joint replacements.^1^ Theoretically, treating OA by stabilizing or reversing the articular cartilage damage would reduce pain and improve patient function, potentially negating the need for joint replacements and, thus, reducing the burgeoning social and economic cost of joint replacements in an ever-aging population that increasingly expects greater levels of physical activity and mobility.^2–5^

Cost-effective noninvasive methods for evaluating tissue quality and regeneration following articular cartilage treatments are not yet available. The existing clinical diagnostic methods have difficulty in detecting early osteoarthritic changes and cartilage lesions.^6^ Clinical assessment of OA is indirect, relying on symptoms and signs of stiffness, pain, swelling, and muscle weakness. Imaging of joints with plain x-ray to assess joint space narrowing, the appearance of subchondral bone sclerosis, and the presence of osteophytes is cheap and simple but unsuitable for early diagnosis as the cartilage is not imaged directly.^7–9^ Magnetic resonance imaging (MRI) and computed tomography (CT) imaging showed early promise for detection of OA and assessment of cartilage treatments but have proven to be limited in resolution or quantitative capability for detecting early OA.^10,11^ Thus, quantitative imaging of an *in situ* biomarker of cartilage health remains the most desirable strategy.^12^

**FIG. 1.**
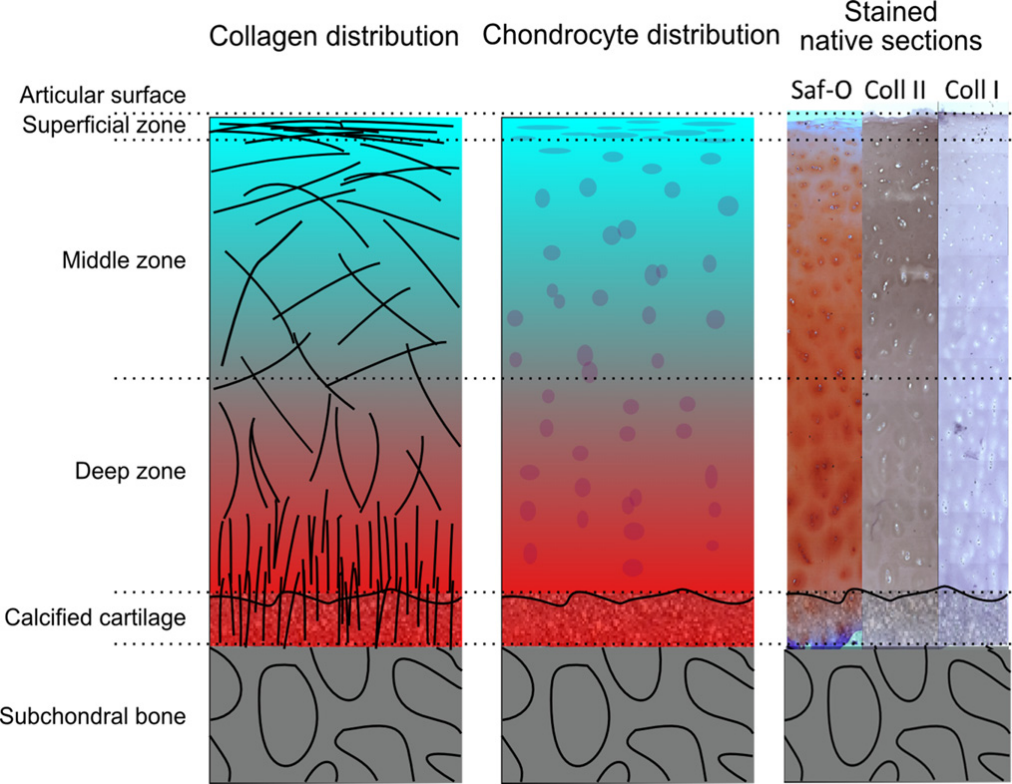
Schematic overview of healthy articular cartilage showing the distributions and zonal organization of collagen and chondrocytes, as well as gradients in GAG, Collagen I, and Collagen II content via histology (Safranin-O) and immunohistology (Coll II, Coll I) staining.

Sulfated glycosaminoglycans (sGAG or GAG) are sensitive biomarkers of early cartilage degradation.^13^ Healthy articular cartilage consists largely of an extracellular matrix containing chondrocytes with water (65–80%), collagens (10–20%), and proteoglycans (10–20%) and can be organized into superficial, mid, and deep zones (Fig. 1).^7,14^

Proteoglycans contain negatively charged GAGs, which form non-covalent interactions with water contributing to the compressive stiffness and lubrication between cartilage surfaces.^15^ The density and sulfation of GAG are highest in the deep zone and decrease in the more superficial layers (Fig. 1), causing a gradient in the negative fixed charge density of the cartilage.^16^ The integrity and physical properties of the articular cartilage are mainly regulated by specialized cartilage cells, the chondrocytes.^17,18^ It is thought that the chondrocytes fail to maintain healthy cartilage in OA due to multifactorial influences including biomechanical, biochemical, and immunological events.^19,20^

The exact etiology of OA remains unknown despite several risk factors having been identified.^19,21,22^ What is known is that the GAG content of articular cartilage reduces following damage and increases after successful repair and regeneration.^23–25^ GAG can be evaluated via histological methods, using Safranin-O or Toluidine blue staining, or measured by biochemical methods such as the dimethylmethylene blue (DMMB) assay.^18,25,26^ While both histological and biochemical assay techniques represent the gold standard for effective detection, histological scoring, and quantitative assessment of GAG and tissue quality, they are, nonetheless, destructive and invasive, requiring the harvesting of a biopsy of cartilage tissue from the joint. For this reason, minimally invasive MRI and CT imaging techniques have been developed for semi-quantitative assessment of the GAG content in both clinical and research settings.^27–29^ For example, previous noninvasive imaging techniques have exploited the negative charge exhibited by GAGs by using anionic contrast agents based on iodine (I) or gadolinium(Gd).^29–36^ These negatively charged contrast agents (ioxaglate containing iodine for CT and gadobenate dimeglumine (Gd-BOPTA) containing gadolinium for MRI) are introduced either directly (intra-articular) or systemically (intravenous). The negatively charged GAG subsequently displaces the negatively charged contrast agent, resulting in regions where the high signal or attenuation of the contrast agent reflects regions of low GAG concentration and vice versa. Consequently, this inverse relationship between the contrast agent and the GAG content offers the potential to nondestructively image changes in the GAG concentration evident in early OA by detecting differences in the uptake of a charged contrast agent.

Anionic ioxaglate (Hexabrix^®^ 320, Guerbet, France), an iodine-based contrast agent, is useful as an inverse indicator for GAG used in CT. The micro-CT (*μ*CT) technique is known as Equilibrium Partitioning of an Ionic Contrast agent (EPIC) *μ*CT.^37–40^ EPIC-*μ*CT has demonstrated imaging of GAG distribution; however, it cannot discriminate between materials of similar x-ray attenuation. For example, in OA, the articular cartilage adjacent to the bone may be depleted of GAG and, therefore, exhibits high concentrations and attenuation of the anionic contrast agent, and EPIC-*μ*CT has difficulty in distinguishing the two. Since changes in the subchondral bone are another marker of OA, it is highly desirable to measure levels of GAG and the features of the adjacent subchondral bone concurrently.^41–43^

An alternative imaging modality, Delayed Gadolinium-Enhanced MRI of Cartilage (dGEMRIC), uses MRI to measure the relaxation of hydrogen atoms in cartilage bathed in a paramagnetic gadolinium contrast agent, for example, gadobenate dimeglumine (MultiHance^®^, Bracco, USA).^29^ This imaging technique is sensitive for cartilage degradation, but is costly, does not have sufficiently high spatial resolution to assess thin layers of cartilage, and has poor visualization of subchondral bone features.^7,37^ Furthermore, there remain conflicting evidence for its value in early OA and doubts that it correlates well with clinical measures of cartilage repair.^44,45^

MARS Photon-processing spectral CT has been shown to discriminate between two highly attenuating materials based on their spectral signatures in other applications.^32,46^ Photon-processing spectral CT uses a photon-counting detector to discriminate and quantify multiple materials simultaneously, which is not possible using the energy-integrating detectors found in EPIC-*μ*CT or conventional full body clinical CT.^30,34^ MARS photon-processing spectral CT analyzes the energy-dependent characteristic x-ray spectra of different materials, to create a ‘color’ image of the object of interest, which we have previously shown can provide both structural and material-related information in the same image.^32,34,35^

In this study, we aimed to demonstrate that photon-processing spectral CT imaging could spatially locate and quantify the GAG content using multiple commonly used contrast agents (through an inverse relationship with ioxaglate and/or gadobenate dimeglumine), yet clearly differentiates cartilage from bone. We sought to test this aim via the systematic investigation of quantitative photon-processing spectral CT imaging of GAG distribution in healthy bovine tissue with both iodine- and gadolinium-based contrast agents validated against quantitative biochemical analysis (DMMB assay) of the GAG content as well as histological (Safranin-O) assessment. Furthermore, as proof of concept for nondestructive clinical imaging of GAG distribution and quantitative assessment of cartilage health and bone architecture, we investigated photon-processing spectral CT of osteoarthritic human tibial plateau samples from patients undergoing total joint arthroplasty.^14,29,36,40^

## RESULTS

### Measurement of GAG in healthy cartilage

**FIG. 2.**
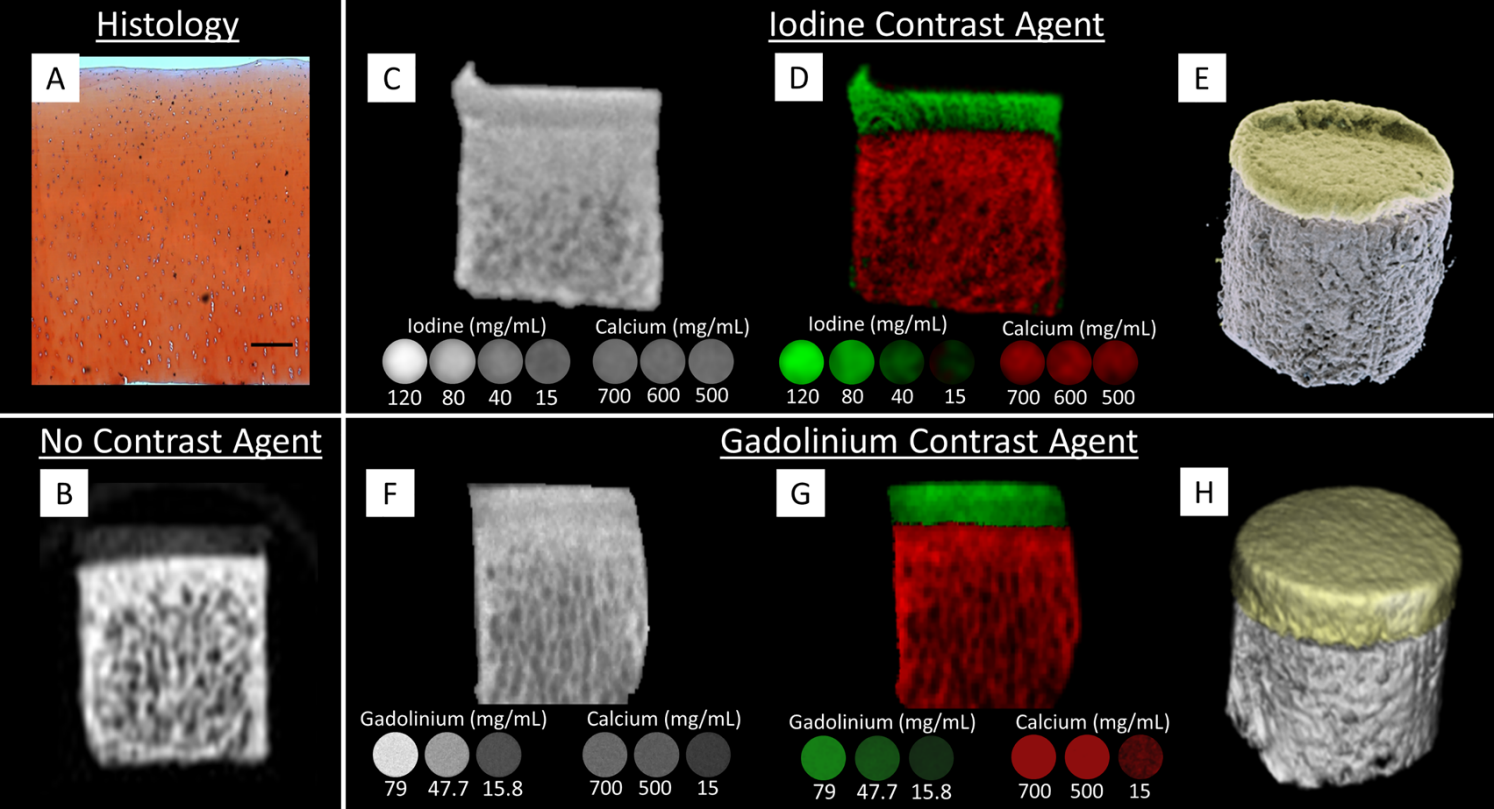
(a) Histological staining (Safranin O/hematoxylin/fast green) of GAG distribution in full thickness healthy bovine cartilage (scale bar = 200 *μ*m). (b) Cartilage region in the cartilage-bone core not incubated in the contrast agent is hardly visible and no gradient can be observed; however, bone is highly attenuating. (c) and (f) Reconstructed image of the cartilage-bone core scanned with MARS following incubation in ioxaglate or gadobenate dimeglumine, respectively. Concurrently imaged calibration tubes with varying concentrations of iodine, gadolinium, and calcium are included below the sample image. (d) and **(**g) False-color materially decomposed MARS image of the same sample and corresponding calibration tubes, in panels **(**c) and **(**f), with iodine and gadolinium represented in green and calcium shown in red. (e) and **(**h) 3D rendering including the sample composition with calcium in white and iodine or gadolinium in yellow. The sample is 8 mm in diameter.

Ioxaglate and gadobenate dimeglumine reached (near) diffusion equilibrium within 24 h as no statistical difference was found between the 24 h and 48 h measurements of both iodine and gadolinium concentrations, with respective *p*-values of 0.710 and 0.957. MARS material maps of cartilage-bone cores incubated in ioxaglate and gadobenate dimeglumine exhibited a gradient of attenuation through the cartilage. The cartilage surface was highly attenuating, with decreasing attenuation until the bone was reached. Alternatively, cartilage on cores without the contrast agent was much less visible with no attenuation difference seen through the cartilage. The expected gradient in the GAG concentration was confirmed with histology showing GAG concentrations, red color from Safranin O staining, low near the top, and high near the bottom. Therefore, an inverse relationship between attenuation and GAG distribution could be identified in the MARS images of the samples (Fig. 2). In both cases, with and without the contrast agent, the bone was highly attenuating and bone porosity was visible. The material decomposition images of the cartilage-bone cores illustrate a clear distinction between iodine/gadolinium within the cartilage (false colored with the heat map) and calcium in the underlying bone.

**FIG. 3.**
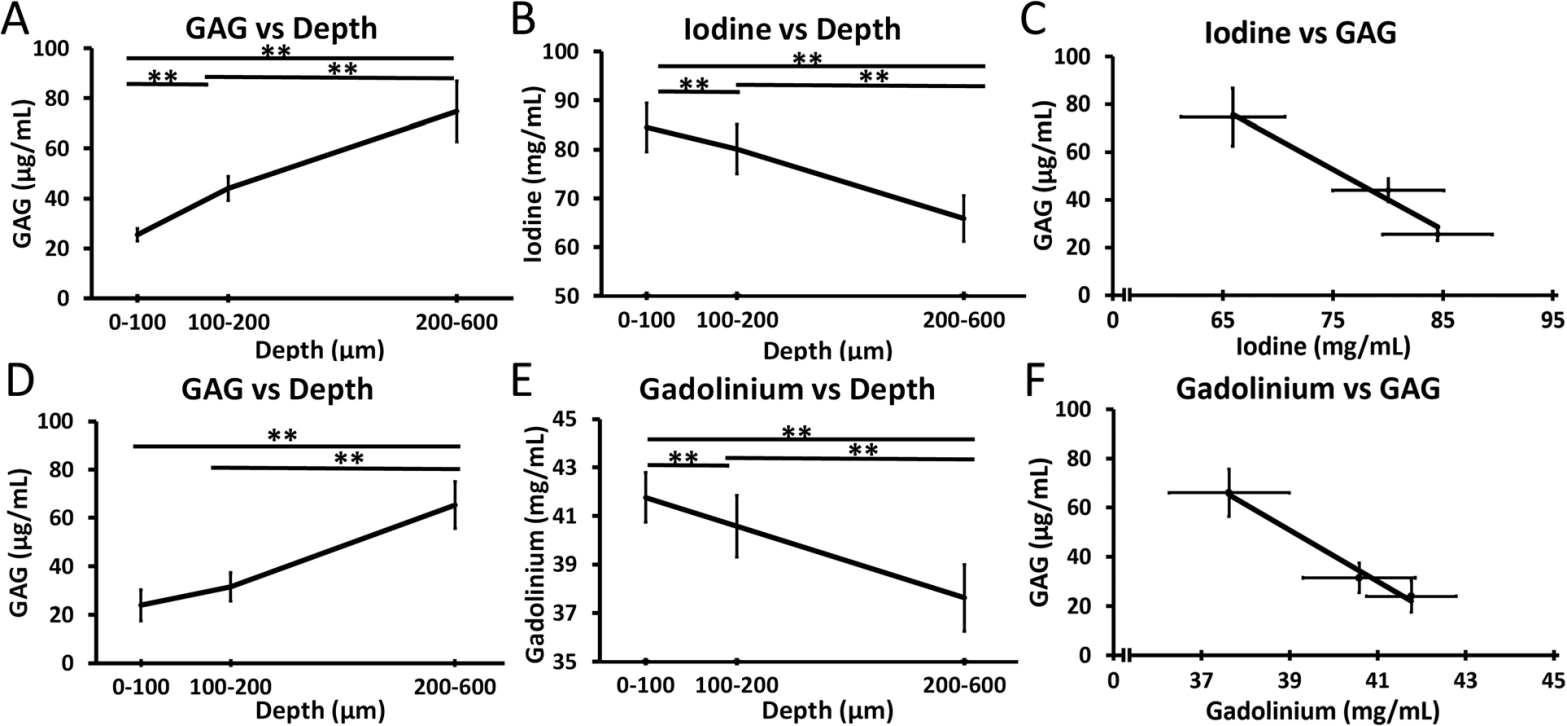
(a) Average concentration of the GAG content in cartilage (n = 4) increases with the depth into the deep zone cartilage. (b) Concentration of iodine at different depths in healthy bovine cartilage (n = 4) as measured by MARS decreases with the increasing depth into the cartilage. (c) GAG plotted against iodine concentrations illustrating an inverse relationship. (d) Average concentration of GAG in the cartilage (n = 4) for a separate set of samples from a second stifle joint (e) Concentration of gadolinium at different depths in healthy bovine cartilage (n = 4) as measured by MARS decreases with the increasing depth into the cartilage. (f) GAG plotted against gadolinium concentrations illustrating an inverse relationship. Data are presented as mean ± SE; ** indicates *p *<* *0.001.

The concentration of iodine and gadolinium in the cartilage was determined by measuring the attenuation from MARS images and fitting this value to an attenuation-concentration curve, based on known calibration phantom values. The concentrations of iodine and gadolinium had an inverse relationship to the biochemical measure of GAG using DMMB assay. The connection between the measured attenuation, concentration of iodine or gadolinium, and GAG concentration allowed for the determination of GAG in the cartilage based on the measured iodine/gadolinium concentration in MARS images. Both iodine and gadolinium concentrations had a linear relationship to attenuation. MARS imaging measured an average iodine concentration of 85 mg/ml corresponding to an average GAG concentration of 25 *μ*g/ml from DMMB assay samples in the cartilage superficial layer. For deep layer quantification, MARS imaging measured an average iodine concentration of 65 mg/ml corresponding to an average GAG concentration of 75 *μ*g/ml from DMMB assay samples. MARS imaging measured an average gadolinium concentration of 43 mg/ml corresponding to an average GAG concentration of 30 *μ*g/ml from DMMB assay samples in the superficial layer. In the cartilage deep layer, MARS imaging measured an average gadolinium concentration of 37 mg/ml corresponding to the average GAG concentration of 75 *μ*g/ml from DMMB assay samples (Fig. 3). Quantitative biochemical data of GAG measured by destructive sampling and DMMB assay and quantitative MARS imaging data of the iodine concentration comparing superficial and deep layers of the articular cartilage support these observations, with significantly higher (*p *<* *0.05) levels of iodine content in superficial cartilage and a significantly greater quantity of GAG in the deep zone (*p *<* *0.05). This matches the expected distribution of GAG in healthy bovine articular cartilage and was also observed in the histological sections.^54^ Likewise, similar results were seen in cores with gadolinium concentrations. The GAG content for these cores showed an overall increase with the increasing depth; however, GAG concentrations in superficial and middle layers were not statistically significantly different (*p *=* *0.14).

**FIG. 4.**
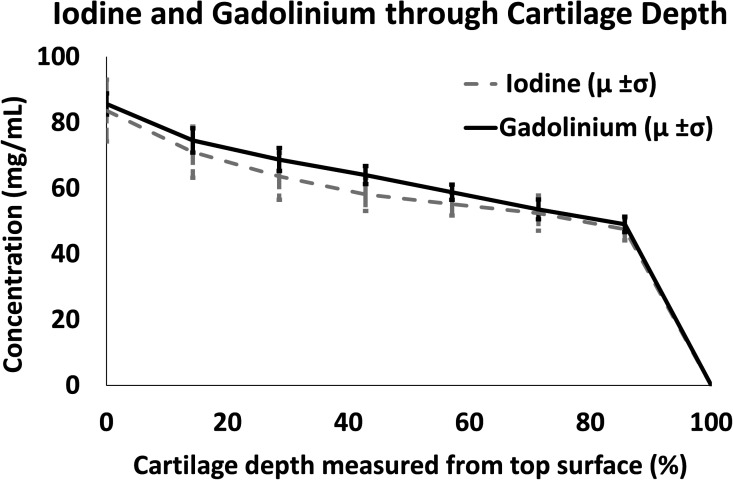
Measurement of iodine and gadolinium concentrations throughout the thickness of cartilage, approximately 800 *μ*m (from superficial (0%) to the deep zone (100%)), in the same sample of healthy tissue based on attenuation of the known concentration in calibration phantoms. Data are presented as mean ± SD.

Comparison of the two contrast agents (ioxaglate and gadobenate dimeglumine) in healthy bovine tissue (Fig. 4) demonstrated that the profile of uptake through the depth of the cartilage was similar for both contrast agents. The similar uptake in concentrations of ioxaglate (iodine) and gadobenate dimeglumine (gadolinium) in the same sample indicates that both agents are sensitive to the GAG gradient observed in cartilage and are able to measure similar concentrations of GAG. No difference was observed based on the order of incubation.

### Photon-processing spectral CT imaging of osteoarthritic cartilage

**FIG. 5.**
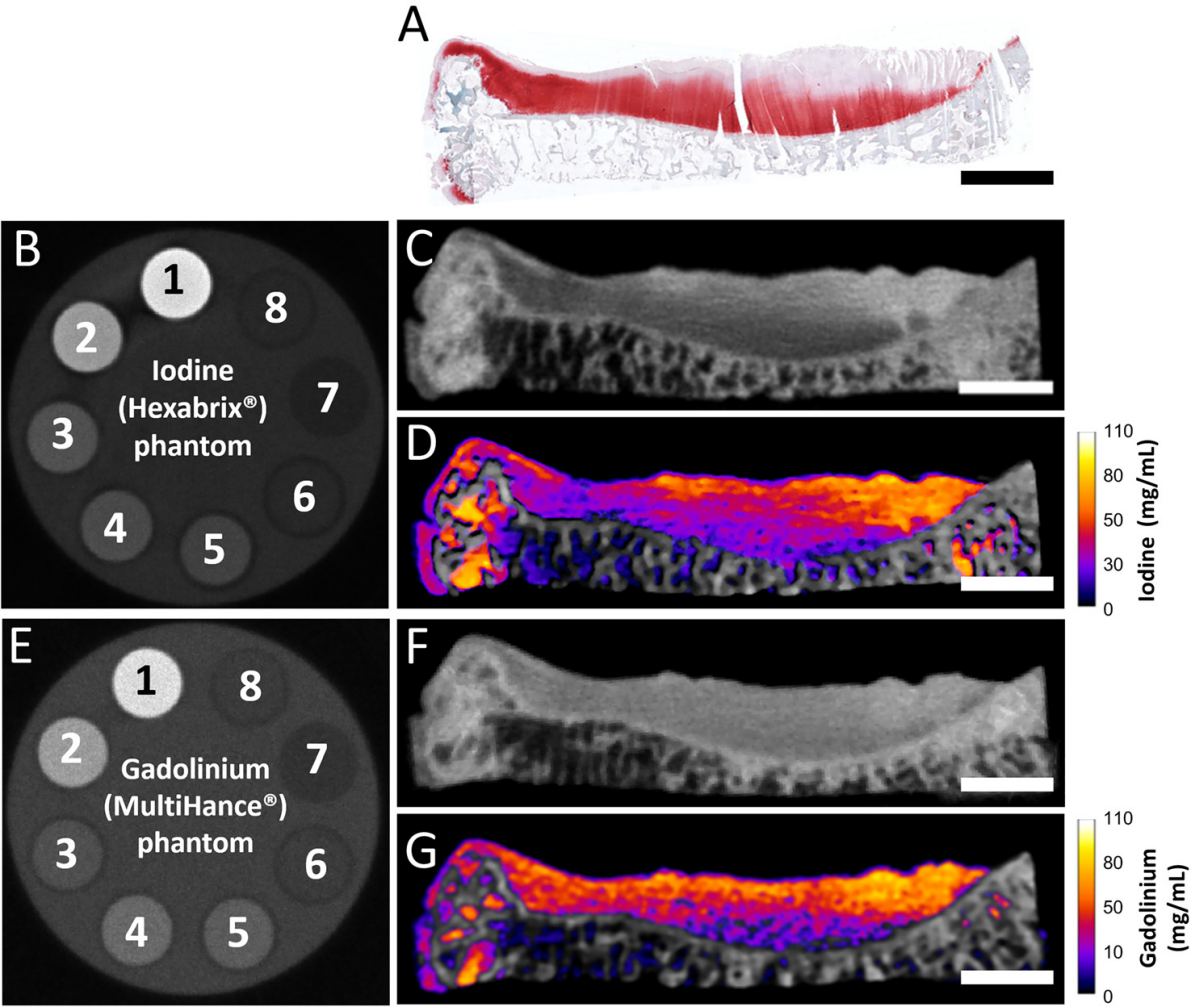
(a) Histological image of a lateral human tibial plateau, stained with Safranin O/fast green/hematoxylin. Red color from Safranin O stain indicates the presence of GAG. (b) Iodine phantom (1: 160 mg/ml I; 2: 96 mg/ml I; 3: 32 mg/ml I; 4: 700 mg/ml CaCl_2_; 5: 500 mg/ml CaCl_2_; 6: 15 mg/ml CaCl_2_; 7: Oil; 8: water). (c) Single-energy image (to mimic a conventional CT image) slice of the sample incubated in ioxaglate. (d) Material-decomposed MARS image obtained from the same sample shown in panel A. The image shows the concentration of iodine from the anionic contrast agent (shown in the heat map as per bar on right). Calcium is shown on the grayscale. (E) Gadolinium (Gd) phantom (1: 79 mg/ml Gd; 2: 47.4 mg/ml Gd; 3: 15.8 mg/ml Gd; 4: 700 mg/ml CaCl_2_; 5: 500 mg/ml CaCl_2_; 6: 15 mg/ml CaCl_2_; 7: Oil; 8: water). (f) Single-energy image (to mimic a conventional CT image) slice of the sample incubated in gadobenate dimeglumine. (g) Material-decomposed MARS image obtained from the sample shown in panel A. The image shows the concentration of gadolinium from the anionic contrast agent (shown in the heat map as per bar on right). Calcium is shown on the grayscale. Scale bars indicate 5 mm. Note: purple layer on the top articulating surface of cartilage (d) and (g), indicating the low Gd (high GAG) concentration, is an imaging artifact due to the partial volume effect.

In sections of OA samples not completely denuded of cartilage, some GAG was still visible in the residual articular cartilage; however, these samples had a significantly different appearance based on the distribution of contrast agents from healthy cartilage. The MARS material-decomposed images (Fig. 5) again showed an inverse correlation between iodine [Fig. 5(d)] and gadolinium contrast [Fig. 5(g)] with the GAG content observed in histological images [Fig. 5(a)], with high attenuation in the superficial zones of the cartilage where the histological images displayed reduced GAG staining. In regions of severely degenerated cartilage, there was a lack of GAG throughout the majority of thickness of the cartilage, in addition to morphological changes in the tissue. Material decomposition was able to separately identify the contrast agent (iodine or gadolinium) and the calcium present in subchondral bone. In the lateral tibial plateau, even when increased concentrations of highly attenuating contrast agent are present directly adjacent to the bone, a clear distinction could be observed between cartilage and bone.

## DISCUSSION

This study analyzed whether photon-processing spectral CT can spatially locate and quantify the GAG content in healthy articular cartilage and osteoarthritic human articular cartilage using iodine- and gadolinium-based contrast agents, validated against destructive biochemical assay and histological techniques. There are five key findings in this study. First, photon-processing spectral CT can accurately determine iodine and gadolinium levels in articular cartilage incubated in ioxaglate and gadobenate dimeglumine. Second, from quantification using MARS imaging, anionic iodine and gadolinium concentrations have a strong inverse relationship to the GAG content. Third, nondestructive photon-processing spectral CT with ioxaglate or gadobenate dimeglumine correlates strongly with histological images of cartilage stained for GAG. Fourth, photon-processing spectral CT can distinguish healthy articular cartilage high in GAG content from unhealthy cartilage low in GAG content. Finally, ioxaglate or gadobenate dimeglumine in the deep zone articular cartilage can be easily distinguished from subchondral bone exhibiting similar attenuation levels.

These results suggest that photon-processing spectral CT offers significant clinical applicability in the future as a nondestructive, single-imaging modality for determining the health of multiple tissue types. This is particularly relevant considering that the most direct method to currently measure cartilage quality is to obtain histological specimens with biochemical assays, which is destructive and invasive to patients as well as risking further tissue degeneration and morbidity from biopsy harvest. Nondestructive imaging modalities are, therefore, preferred, but noninvasive assessment of cartilage remains challenging.^54^ To date, MRI has provided the best soft tissue contrast, but has a lower spatial resolution than CT. Furthermore, CT is available to nearly all patients, whereas MRI has several contraindications and is not available to most patients with metallic implants.^55^ In contrast, photon-processing spectral CT provides high resolution three-dimensional (3D) images where different materials can be identified and quantified. Our results demonstrated that photon-processing spectral CT can accurately and simultaneously assess zonal GAG distribution in cartilage using multiple contrast agents, Hexabrix^®^ (for CT) and MultiHance^®^ (for MRI), as well as identify and separate calcium in underlying bone for assessment of both cartilage and bone health.

Of particular interest are the material decomposition images of the bone-cartilage explants, which demonstrated a clear distinction between the cartilage and the underlying bone (Fig. 2). This distinction contrasts other imaging modalities, including EPIC-*μ*CT, where the highly attenuating ioxaglate in the deep cartilage is often difficult to distinguish from the highly attenuating subchondral bone.^37^ As EPIC-*μ*CT relies on attenuation differences to distinguish iodinated contrast from bone, a reduced concentration of ioxaglate is selected for EPIC-*μ*CT to ensure that the cartilage can be distinguished from bone.^38^ Our results demonstrate that using MARS imaging allows for a clear distinction between bone and cartilage to be identified.

The separation of bone and cartilage has also been addressed in other studies by the development of cationic contrast agents used alone or in combination with low concentrations of iodine-based contrast agents.^15,56^ Cationic contrast agents have a direct relationship to the GAG content and allow clearer visual separation from subchondral bone. Utilizing a combination of agents with different diffusion driving forces has been theorized as a more sensitive technique for quantification of the GAG content. However, when cartilage health is degraded and the GAG content is low, the sensitivity of cationic agents decreases.^57,58^ This is of particular importance in the superficial layer for healthy cartilage where the low GAG concentration and/or low GAG sulfation (i.e., low fixed charge density) is not as sensitive to the inverse relationship demonstrated in this study as with cationic agents.^16^ However, the ability to distinguish iodine, gadolinium, and calcium using photon-processing spectral CT means that ioxaglate and gadopentetate dimeglumine concentrations can be selected to maximize the sensitivity of the measurement, rather than being constrained by the need to differentiate the contrast agent from bone. Furthermore, using a higher concentration of contrast agent with photon-processing spectral CT will provide increased sensitivity of GAG quantification.

**FIG. 6.**
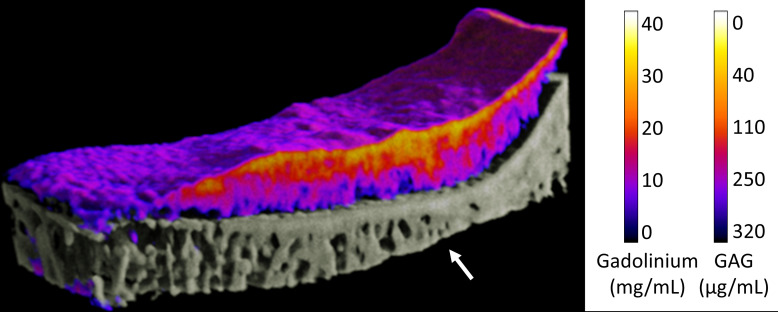
Cross-sectional 3D rendering of osteoarthritic human tibial plateau incubated in the gadolinium (50% MultiHance^®^) contrast agent. Cartilage quality is quantified based on the GAG content validated from biochemical analysis (see the heat map) as well as 3D GAG distribution (medial-lateral; posterior-anterior), whereas bone quality is assessed based on 3D structural properties and density, all within the single imaging modality. The GAG content shown here is based on linear relationships determined in Fig. 3 for gadolinium. The reduced GAG content (yellow/orange) and sclerotic bone (arrow) are key factors of OA progression. Note: the purple layer on the top articulating surface of cartilage, indicating the low Gd (high GAG) concentration, is an imaging artifact due to the partial volume effect.

Clinical translation and validation of cationic, dual contrast imaging, and photon-processing spectral CT for OA evaluation are under investigation.^59,60^ For clinical translation of photon-processing spectral CT, it is recognized that incubation periods of 24h utilized in this study are not practical and optimization of *in vivo* incubation times, concentrations, and delivery is needed.^59^ Shorter incubation times, necessary for clinical use, would not significantly affect attenuation characteristics and material decomposition as a similar uptake in the tissue would be expected with a majority of diffusion occurring initially.^11^ An additional challenge in translation noted in this study was the partial volume effect. However, this effect has been shown to be reduced when samples are imaged in liquid and, thus, will be reduced when translated to *in vivo* imaging with the presence of synovial fluid, while presenting the new challenge of separation of the superficial layer of cartilage from the contrast agent in the knee capsule. Another point in clinical translation for photon-processing CT is that imaging of both iodine and gadolinium based contrast agents with the same modality is suitable for clinical use as patients with varying conditions (i.e., kidney disease, allergic reactions to certain agents. etc.) would be able to undergo imaging and assessment with the same resolution, radiation exposure, and information obtained.^60^ Overall, the ability to distinguish both tissues using photon-processing spectral CT provides a foundation for future work in quantitative 3D assessment of both cartilage and bone health and OA progression from a single scan (Fig. 6).

The volume of research into the diagnosis and management of early OA continues to mount.^61,62^ Despite the lack of successful long-term treatments or regenerative strategies available clinically to arrest or reverse early OA,^54,63^ the need for accurate, nondestructive and quantitative imaging technologies to rapidly diagnose and assess tissue health continues to accelerate.^64–67^ Photon-processing spectral CT imaging, therefore, offers the researcher or clinician the capacity to monitor early onset OA and/or the articular cartilage response to new OA treatments,^62^ as well as study the progression of tissue-engineered cartilage or bone repair tissues *in vivo* over time in human or pre-clinical animal models. One of the key outcomes of this study was validation of photon-processing spectral CT and associated material decomposition of the gadolinium contrast agent as a single imaging modality to quantify the GAG concentration in *μ*g/ml throughout full thickness cartilage (Fig. 6) relative to destructive biochemical assay (DMMB) of the GAG content, as well as provide 3D structural information on subchondral bone. This combination of detection of GAG depletion, an early biomarker of OA, as well as subchondral bone changes provides a quantitative overview of joint changes as a whole. It is important to note that human tibial plateaus examined in this study harvested following TKA had visual signs of early OA on the lateral side. However, further investigation is needed and under way to validate the ability of photon-processing spectral CTs to distinguish healthy, early, and late OA in both cartilage and bone. Detection and quantification of changes in subchondral bone (e.g., evidence of sclerosis or osteochondritis dissecans) especially in combination with the reduced GAG content are key factors for identification of OA progression and diagnosis of early onset OA. The ability of photon-processing CT to quantitatively measure cartilage and bone composition and morphology at a high resolution is ideal for OA detection and allows imaging of joint changes and overall joint homeostasis.

One of the limitations of this study is related to photon-processing spectral CT outcomes on human tissue explants post-TKA surgery for comparison with existing human clinical imaging. However, we believe that this study demonstrates a proof of concept and we are currently undertaking a larger study to investigate clinical MRI and photon-processing spectral CT in an OA patient cohort. Another limitation is the use of bovine cartilage as a surrogate for healthy human cartilage. This tissue was selected to be used because of the challenges in acquiring sufficient healthy human articular cartilage tissue. Importantly, due to its similar thickness to human articular cartilage (approximately 2–3 mm^68–70^), bovine articular cartilage tissue was selected in order to optimize imaging parameters and validate the contrast agent diffusion and biochemical GAG content, for direct translation to human photon-processing spectral CT studies.^71^ The scans were performed in the human energy range, using a spectral detector platform being scaled to meet human scanning needs.^72^ Furthermore, evidence for clinical translation pathways and contrast agent delivery includes recently established methods for intra-articular injection of gadoterate meglumine (Gd-DOTA; Dotarem^®^, Guerbet, France) in a pig OA model^62^ and current methods for administering gadolinium contrast agents clinically via intravenous injections adopted for dGEMRIC.^6^ We believe that these barriers to clinical translation can be overcome and spectral imaging methods have significant potential to image and quantify cartilage and bone health.

## CONCLUSIONS

MARS photon-processing spectral CT imaging can quantitatively determine the quality of both healthy and osteoarthritic articular tissue. Zonal articular cartilage health from MARS imaging correlated with destructive histological examination and quantitative biochemical GAG assays of articular cartilage, including accurately determining the GAG content present in full-thickness cartilage layers. Statistically significant differences were observed in the GAG content in zonal layers of the cartilage, which had an inverse relationship with both iodine- and gadolinium-based contrast agent concentrations present in normal tissue. Furthermore, photon-processing spectral CT provides a high-resolution, nondestructive method for quantification of GAG in healthy and OA human cartilage explants and is able to distinguish two adjacent high-contrast materials based on their spectral signatures, enabling a clear distinction between identification of contrast agents absorbed by cartilage and bone.

## METHODS

### Healthy cartilage: Sample preparation

Cylindrical healthy cartilage-bone cores (8 mm diameter; n = 42 total) were obtained using a core drill from the femoral condylar surface of bovine stifle joints. Cartilage bore cores collected for direct comparison of biochemical (n =8) and histological (n = 8) evaluation with contrast enhanced MARS imaging (n = 8) were taken adjacent to each other on the same stifle joint. Cartilage-bone cores without the contrast agent (n = 4) were used as negative controls. A separate set of samples (n = 14) was used for the MARS imaging assessment of incubation periods and uptake of both agents, Hexabrix^®^ and MultiHance^®^ in the same sample. All samples imaged using MARS were fixed in 10% formalin for 48 h, which has been shown to have no significant effect on the GAG concentration.^40^ While it has been noted that formalin fixation may reduce cartilage attenuation with *μ*CT, this result was not observed in preliminary work with photon-processing spectral CT.^47^ All samples for histology and biochemical analysis only included the cartilage, which were sharply dissected from the bone. Samples for histological analysis were fixed in 10% formalin for 48 h and subsequently paraffin embedded and sectioned into 4 *μ*m slices. For biochemical analysis, the cartilage was core biopsied with a punch (7.80 mm diameter). The samples were then frozen in the Optimal Cutting Temperature mounting media compound (VWR, East Grinstead, UK), and serial sections were cut from the articular surface to the deep zone of the cartilage using a Leica CM1510 Cryostat Microtome (Leica Biosystems, Nussloch, Germany) at 20 °C. Sections from each core were pooled to obtain samples for each of the zones defined as superficial: 0–100 *μ*m, middle: 100–200 *μ*m, and deep: 200–600 *μ*m.^48^

### Incubation parameters

The incubation solutions consisted of phosphate buffered saline (PBS), protease inhibitors (Sigma-Aldrich P2714, 1X stock concentration), 0.01% sodium azide (*NaN*_3_), and Hexabrix^®^ or MultiHance^®^. A concentration of 80 mg I/ml (50% Hexabrix^®^ + 50% PBS) was used for iodine incubation (n = 4). A concentration of 79 mg Gd/ml (100% MultiHance^®^) was used for gadolinium incubation (n = 4). Concentrations of contrast agents were based on previous experiments described by Rajendran *et al.*^35^ Incubation was performed at 37 °C under continuous rotation with the sample fully submerged in solution. The time required to reach diffusion equilibrium of contrast agents was investigated for Hexabrix^®^ (n = 4) and MultiHance^®^ (n = 4) by performing scans after 24 h and 48 h of incubation. Distribution of both agents in the same sample was investigated with 24 h incubation in the first agent, a 24 h washout period, 24 h incubation in the second agent, and a 24 h washout period. Order of incubation was accounted for by using Hexabrix^®^ first (n = 3) for one group and MultiHance^®^ first (n = 3) for another. Prior to MARS imaging, the samples were rinsed with PBS for 30 s to rinse excess contrast agent from porous bone regions.

### Photon-processing spectral CT data acquisition and image processing

Cartilage-bone cores were placed inside a tube, along with PBS-soaked cotton wool to provide a humidified environment during scanning. A MARS-CT scanner (MARS Bioimaging Ltd, Christchurch, New Zealand) equipped with a 2 mm CdTe-Medipix3RX camera with a pixel size of 110 *μ*m was used to scan the samples. The settings are outlined in Table I.

**TABLE I.** MARS photon-processing spectral CT imaging protocol for healthy cartilage. Different energy bins were used for iodine (Hexabrix^®^) and gadolinium (MultiHance^®^) for identification of the element K-edge. Scans were performed using Charge-Summing Mode to assign shared photon counts to the correct pixel.

Contrast agent	Ioxaglate (Hexabrix^®^)	Gadobenate dimeglumine (MultiHance^®^)
kVp	80	120
Tube current	80 *μ*A	30 *μ*A
Exposure time	70 ms	130 ms
Source object distance	166.2 mm	160 mm
Object detector distance	84 mm	100 mm
Energy bins (N)	4	4
Energy thresholds (keV)	20, 28, 35, 40	27, 33, 49, 60

Calibration tubes containing known concentrations of potassium iodide (KI: 15, 40, 80, and 120 mg/ml), gadobenate dimeglumine (Gd: 15.8, 47.4, and 79.0 mg/ml), and calcium chloride (CaCl_2_: 15, 500, 600, and 700 mg/ml) were used as references to quantify iodine, gadolinium, and calcium contents in the cores using MARS imaging.

The raw data from the specimen scans were reconstructed using MARS Bioimaging’s iterative reconstruction algorithm.^49^ ImageJ software (National Institutes of Health, USA) was used to measure the linear attenuation coefficients due to iodine and gadolinium. Regions of interest (ROIs) were selected for the three zones of the cartilage (superficial: top 100 *μ*m; middle: 100–200 *μ*m; deep: 200–600 *μ*m), which corresponded to the histological and biochemical results. Image artifact, most notably the partial volume effect, was accounted for in ROI selection by starting one voxel below the superficial articulating surface of the cartilage. The spectral profiles (energy-dependent X-ray attenuation profiles) of the different materials in the calibration tubes were used to spatially identify iodine, gadolinium, and calcium in the cartilage-bone samples, using the material decomposition technique described by Batemen and colleagues.^50^

For diffusion equilibrium and uptake in the same sample analysis, after material decomposition was completed, the concentration of the contrast agent was measured perpendicular on the cartilage surface from the subchondral bone to the superficial cartilage layer across >70% of the total cartilage volume. The average concentrations of the plugs at 24 h and 4 8h were compared. Additionally, the average iodine concentration at 24 h was compared with the average gadolinium concentration at 24 h. For both comparisons, ANOVA was used as a statistical method with a selected significance threshold of *p *<* *0.05.

### Histology

Following fixation, dehydration, and paraffin embedding and sectioning, the samples were stained with 0.1% w/v Safranin-O (Sigma-Aldrich, India), 0.001% Fast Green FCF solution (Sigma-Aldrich, UK), and Gill’s hematoxlin (Merck, USA) for GAG identification. These samples were imaged using a Carl Zeiss Axio Imager 2 microscope (Carl Zeiss Microscopy, Germany) at 5× and 10× magnification.

### Biochemical GAG measurement

To quantify the content of GAG in the samples, the reaction of GAG with dimethylmethylene (DMMB) blue was measured. This technique relies on the absorption spectra of the change in 1,9-dimethylmethylene dye due to the induction of metachromasia when it binds to sulfated GAGs.^51^

Frozen cartilage sections were digested overnight at 56 °C in 500 *μ*l 1 mg/ml proteinase-K solution in digestion buffer (Tris/EDTA buffer). Dimethylmethylene blue chloride (16 *μ*g/ml dimethylmethylene blue) (Sigma-Aldrich, USA) in a solution of 3.04 mg/ml glycine and 2.37 mg/ml NaCl dissolved in 0.01M HCl in d_2_H_2_O (pH 3) was added directly followed by the measurement of the absorbance at 520 nm using a Varioskan Flash Multimode Reader (Thermo Scientific). Chondroitin sulphate-B (Sigma-Aldrich, USA) was used to generate a standard curve, and GAG measurements were normalized to the wet weight.^52^ An ANOVA with a selected significance threshold of *p *<* *0.05 was used to determine statistically significant differences between the GAG content for each layer of cartilage.

### Human osteoarthritic cartilage: Sample preparation

Ethical approval was obtained from the New Zealand Health and Disability Ethics Committee (URB/07/04/014). Written informed consent was obtained from all subjects (patients, n = 1) in this study. Intact tibial plateau samples were obtained during total knee arthroplasty (TKA) surgery following consent and stored for 3 h in PBS. 25 mm strips containing both the articular cartilage and underlying subchondral bone were cut from the central region of the plateau, encompassing the medial and lateral tibial plateaus. These were then separated into their medial and lateral components and frozen in PBS at −20 °C until the time of use, followed by a slow thawing process to room temperature in order to preserve cartilage integrity.^53^

### Photon-processing spectral CT data acquisition and image processing

The lateral sample from the tibial plateau was thawed and then incubated in iodine incubation solution at 37 °C with constant rotation for 24 h prior to scanning. Calibration tubes with known concentrations of ioxaglate (32, 96, and 128 mg/ml iodine), CaCl_2_ (15, 500, and 700 mg/ml), water, and oil were scanned after the sample scan. Immediately following the scan, the sample was placed into PBS for 24 h to allow the contrast agent to fully diffuse out of the tissue. After 24 h, the lateral sample of the tibial plateau was incubated in gadolinium incubation solution at 37 °C with constant rotation for 24 h before scanning. Calibration tubes with known concentrations of gadobenate dimeglumine (15.8, 47.4, and 79.0 mg/ml gadolinium), CaCl_2_ (15, 500, and 700 mg/ml), water, and oil were scanned immediately after the sample scan.

The specimens were scanned using the same MARS spectral CT scanner described earlier. Scan details are outlined in Table II. After the scan, data were reconstructed using an iterative reconstruction algorithm and material decomposition was completed as described above.^49^ After material decomposition was completed, a heat map was produced for iodine and gadolinium concentrations using the maximum concentration at the surface as the end point and the start point as the first value below 5 mg/ml of iodine or gadolinium adjacent to the bone. The inverse relationship to GAG was based on a linear relationship determined in healthy bovine samples and overlaid on the human OA cartilage images for proof of concept as zonal DMMB layer analysis is not practical in non-healthy OA tissue due to its varying thickness, significantly reduced GAG content, and etiology related damage and superficial layer erosion.

**TABLE II.** MARS photon-processing spectral CT imaging protocol for OA tissue. Different energy bins were used for iodine (Hexabrix^®^) and gadolinium (MultiHance^®^) for identification of the element K-edge. Scans were performed using Charge-Summing Mode to assign shared photon counts to the correct pixel.

Contrast agent	Ioxaglate (Hexabrix^®^)	Gadobenate dimeglumine (MultiHance^®^)
kVp	120	120
Tube current	30 *μ*A	30 *μ*A
Exposure time	130 ms	130 ms
Source object distance	160 mm	160 mm
Object detector distance	100 mm	100 mm
Energy bins (N)	4	4
Energy thresholds (keV)	20, 28, 35, 41	27, 33, 49, 60

### Histology

After the 24h washout period was complete, strips of approximately 5 mm width were cut from the sample blocks. These strips were then divided into two parts (approximately 22 mm in length) to be used for histological staining. These strips were decalcified in a solution of EDTA, pH 7.0, for a period of five weeks. After this period, the samples were rinsed with PBS and dehydrated in an ethanol series ranging from 70 to 100% in five steps.^25^ The samples were embedded in paraffin wax, sectioned (5 *μ*m slices), and stained with Safranin O/fast green/hematoxylin as described above.

## Data Availability

The data that support the findings of this study are available from the corresponding author upon reasonable request.
